# Stage-specific fluorescence intensity of GFP and mCherry during sporulation In *Bacillus Subtilis*

**DOI:** 10.1186/1756-0500-3-303

**Published:** 2010-11-14

**Authors:** Geoff P Doherty, Kirra Bailey, Peter J Lewis

**Affiliations:** 1 School of Environmental and Life Sciences, University of Newcastle, Callaghan, NSW 2308, Australia

## Abstract

**Background:**

Fluorescent proteins are powerful molecular biology tools that have been used to study the subcellular dynamics of proteins within live cells for well over a decade. Two fluorescent proteins commonly used to enable dual protein labelling are GFP (green) and mCherry (red). Sporulation in the Gram positive bacterium *Bacillus subtilis *has been studied for many years as a paradigm for understanding the molecular basis for differential gene expression. As sporulation initiates, cells undergo an asymmetric division leading to differential gene expression in the small prespore and large mother cell compartments. Use of two fluorescent protein reporters permits time resolved examination of differential gene expression either in the same compartments or between compartments. Due to the spectral properties of GFP and mCherry, they are considered an ideal combination for co-localisation and co-expression experiments. They can also be used in combination with fluorescent DNA stains such as DAPI to correlate protein localisation patterns with the developmental stage of sporulation which can be linked to well characterised changes in DNA staining patterns.

**Findings:**

While observing the recruitment of the transcription machinery into the forespore of sporulating *Bacillus subtilis*, we noticed the occurrence of stage-specific fluorescence intensity differences between GFP and mCherry. During vegetative growth and the initial stages of sporulation, fluorescence from both GFP and mCherry fusions behaved similarly. During stage II-III of sporulation we found that mCherry fluorescence was considerably diminished, whilst GFP signals remained clearly visible. This fluorescence pattern reversed during the final stage of sporulation with strong mCherry and low GFP fluorescence. These trends were observed in reciprocal tagging experiments indicating a direct effect of sporulation on fluorescent protein fluorophores.

**Conclusions:**

Great care should be taken when interpreting the results of protein localisation and quantitative gene expression patterns using fluorescent proteins in experiments involving intracellular physiological change. We believe changes in the subcellular environment of the sporulating cell leads to conditions that differently alter the spectral properties of GFP and mCherry making an accurate interpretation of expression profiles technically challenging.

## Background

**Figure 1:**
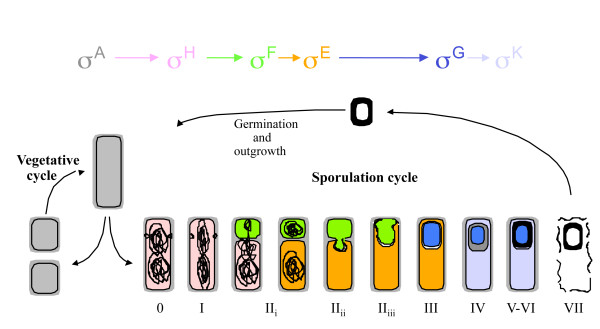
**Schematic of sporulation**. Overview of the sporulation cycle. When vegetative cells encounter conditions of stress such as starvation the sporulation cycle is induced. Division of the vegetative cell occurs asymmetrically forming the mother cell and pre-spore, both containing a copy of the genome. Engulfment of the prespore occurs before the spore coat and cortex are laid down. Eventually the mother cell lyses to release the mature spore. Gene expression is controlled temporally by a subset of sigma factors in both the developing spore and mother cell. The location and time of sigma factor involvement is colour coded in this schematic.

Various Gram positive bacteria can form structures called endospores, which are highly resistant to environmental stress and can remain dormant for thousands of years. The sporulation process can be crudely divided into five stages; Initiation, septation, engulfment, spore and cortex formation and finally maturation and endospore release (Reviewed in [1]). This process is triggered by a stress response such as starvation and results in the expression and repression of a cascade of genes in a tightly controlled temporal manner over several hours in order to form the mature endospore as outlined in Figure 1. After the decision to sporulate has occurred, the rod-shaped cell asymmetrically divides to form a prespore and a much larger mother cell. The mother cell then engulfs the prespore, after which the cortex and the spore coat form. Finally, the mother cell undergoes programmed cell death and the mature endospore is released. This entire process has served as a paradigm for gene regulation and expression and has been extensively studied for over two decades.

We decided to study the recruitment of the transcriptional machinery into the spore during this process using both GFPmut3 and mCherry. The spectral properties of these proteins allows the study of two proteins within the same cell with very little crossover into the other channel [2,3]. During these studies we noticed a trend in fluorescence that was attributable to the fluorescent protein rather than the protein of interest. In this work we present data on the changes in fluorescence emission of GFP and mCherry during the sporulation process, which has wide ramifications on both past and future studies of gene expression and regulation during the sporulation process in *B. subtilis*.

## Materials and methods

### Strain construction growth conditions and image analysis

All plasmids and strains used in this work are detailed in Table 1. GFP cloning was performed by ligation independent cloning (LIC) as detailed in [4] using primers in Table 2. The *mCherry *gene fusions were created by PCR amplifying the 3' end of the respective genes using primers in Table 2, and digesting them with the appropriate restriction enzymes, before ligating them into similarly cut pNG621. Transformation of *B. subtilis *was carried out as per [5]. *B. subtilis *cells were induced to sporulate by the resuspension method of [6] as modified by [7]. Image acquisition and analysis was performed as described by [8].

**Table 1:** Plasmids and strains used in this work

Plasmid	Genotype	Source/Construction
pYG1	*P_spac _-*LIC-*gfpmut3-erm*	[8]

pEU2	*P_spac _-'rpoB-gfpmut3-erm*	This work

pETMCSIII	*bla P_spac _-10-6HisTφ*	[17]

pEU13	*P_spac _-'yloH-gfpmut3-erm*	This work

pEU14	*P_spac _-'ykzG-gfpmut3-erm*	[8]

pEU16	*P_spac _-'rpoE-gfpmut3-erm*	[8]

pEU21	*P_spac _-'rpoC-gfpmut3-erm*	[8]

pEU37	*P_spac _-'nusA-gfpmut3-erm*	This work

pNG583	*bla P_spac _-10-gfpmut3-6HisTφ*	[8]

pNG621	*P_xyl _-*MCS-*mCherry-cat*	[8]

pNG622	*P_xyl_-'rpoC-mCherry-cat*	[8]

pNG670	*P_xyl _-'nusA-mCherry-cat*	This work

pNG677	*P_xyl _-'ykzG-mCherry-cat*	This work

pNG735	*bla P_spac _-10-mCherry-6HisTφ*	This work

**Strain**	**Genotype**	**Source/Construction**

***E. coli***		

BL21(DE3) pLysS	*λ DE3 pLysS; F-ompT{lon}hsdSB (rB--mB-)*	[18]

DH5α	*F- endAI hsdR17 supE44 thi-1 λ- recAI gyrA96 relA1 Δ(lacZY A-argF) U169 Φ80 dlacZ Δμ15*	Gibco BRL

***B. subtilis***		

168trp+	*trpC2 chr::trp*	[4]

EU1	168trp+ *chr:: erm P_wt _rpoB-gfp, P_spac _'rpoB*	This work: 168trp+ transformed with pEU2

EU16	168trp+ *chr:: erm P_wt _yloH-gfp, P_spac _'yloH*	This work: 168trp+ transformed with pEU13

EU17	168trp+ *chr:: erm P_wt _ykzG-gfp P_spac _'ykzG*	This work: 168trp+ transformed with pEU14

EU19	168trp+ *chr:: erm P_wt _rpoE-gfp, P_spac _'rpoE*	This work: 168trp+ transformed with pEU16

EU44	168trp+ *chr:: erm P_wt _rpoC-gfp, P_spac _'rpoC*	[8]

EU49	168trp+ *chr:: erm P_wt _nusA-gfp, P_xyl _'nusA*	This work: 168trp+ transformed with pEU37

EU128	168trp+ *chr:: erm P_wt _rpoC-mCherrry, P_xyl _'rpoC*	This work: 168trp+ transformed with pNG621

EU131	168trp+ *chr:: erm P_wt _ykzG-mCherrry, P_xyl _'ykzG*	This work: 168trp+ transformed with pNG677

EU142	168trp+ *chr:: erm P_wt _nusA-mCherrry, P_xyl _'nusA*	This work: 168trp+ transformed with pNG670

EU156	168trp+ *chr:: cat P_wt _rpoC-mCherrry, P_xyl _'rpoC, erm P_wt _rpoB-gfp, P_spac _'rpoB*	This work: EU126 transformed with pEU2

EU163	168trp+ *chr:: cat P_wt _rpoC-mCherrry, P_xyl _'rpoC, erm P_wt _yloH-gfp, P_spac _'yloH*	This work: EU126 transformed with pEU13

EU164	168trp+ *chr:: cat P_wt _rpoC-mCherrry, P_xyl _'rpoC, erm P_wt _ykzG-gfp, P_spac _'ykzG*	This work: EU126 transformed with pEU14

EU166	168trp+ *chr:: cat P_wt _rpoC-mCherrry, P_xyl _'rpoC, erm P_wt _rpoE-gfp, P_spac _'rpoE*	This work: EU126 transformed with pEU16

EU183	168trp+ *chr:: cat P_wt _rpoC-mCherrry, P_xyl _'rpoC, erm P_wt _nusA-gfp, P_spac _'nusA*	This work: EU126 transformed with pEU37

EU186	168trp+ *chr:: cat P_wt _nusA-mCherrry, P_xyl _'nusA, erm P_wt _ykzG-gfp, P_spac _'ykzG*	This work: EU142 transformed with pEU14

EU224	168trp+ *chr:: cat P_wt _ykzG-mCherrry, P_xyl _'ykzG, erm P_wt _nusA-gfp, P_spac _'nusA*	This work: EU131 transformed with pEU37

EU230	168trp+ *chr:: cat P_wt _ykzG-mCherrry, P_xyl _'ykzG, erm P_wt _rpoC-gfp, P_spac _'rpoC*	This work: EU131 transformed with pEU21

**Table 2:** Primers used in this work

Plasmid	Primer Sequence (5'-3')
pEU2	rpoB F GGGTTCCTGGCGCGAGCGCAGCAGCCTCTTGGCGGTAAAGCGCAATTTGG rpoB R TTGGGCTGGCGCGAGCTTCTTTTGTTACTACATCGCGTTCAACGTCTGC

pEU13	YloH F GGGTTCCTGGCGCGAGCTTAGATCCGTCAATTGATTCTTTAATG YloH R TTGGGCTGGCGCGAGCTTCGCGGTCTTCCTTTTCAAACG

pEU14	YkzG F GGGTTCCTGGCGCGAGCATTTATAAGGTATTTTATCAAGAGAAGGCTG YkzG R TTGGGCTGGCGCGAGCTAACTCCAATACTTTAAAATTTTCGCTCTG

pEU16	RpoE F GGGTTCCTGGCGCGAGCCGCATCTTTGCTCGGCGTG RpoE R TTGGGCTGGCGCGAGCTTTAATTTCCTCTTCTTCATCATCATAGTC

pEU21	RpoC F GGGTTCCTGGCGCGAGCCGTAGAAGTAATGGTTCGCCAG RpoC R TTGGGCTGGCGCGAGCTTCAACCGGGACCATATCGTC

pEU37	NusA F GGGTTCCTGGCGCGAGCCACAGATGATCCTGACGTTGATC NusA R TTGGGCTGGCGCGAGCTTCATCCGATTCAGCAGTTTCAGG

pNG670	NusA mCherry F AAGGGGGGAGACCTCGAGATGAGCAGTGAA (*Xho*I) NusA mCherry R ACCTAAAGTCACGAATTCTTCATCCGATTC (*EcoR*I)

pNG677	YkzG mCherry F AGATTTGGTACCATTTATAAGGT (*Acc*651) YkzG mCherry R TCATACCTCGAGTAACTCCAATA (*Xho*I)

pNG735	pETmCherry F TTTTTTCATATGGTGAGCAAGGGCGAGG (*Nde*I) pETmCherry R TTTTTTGAATTCCTACTTGTACAGCTCGTCC (*EcoR*I)

Restriction sites underlined

### Overproduction and purification of GFP and mCherry

GFPmut3 was overproduced and purified as detailed in [8]. The gene encoding *mCherry *was PCR amplified off pNG621 using pETmCherryF and pETmCherryR (Table 2) and cloned into pETMCSIII using *Nde*I and *Eco*RI to give rise to pNG735 (Table 1). Overproduction, purification and quantification of the purified mCherry protein was carried out as per GFPmut3 as detailed in [8].

### Determining the pH-dependent emission of GFP and mCherry

The pH of potassium phosphate buffers (20 mM KH_2_PO_4_, 200 mM NaCl, 10% glycerol) were adjusted using either 5 M KOH or 5 M HCl to yield twelve buffers with a pH of 4.4, 5.0, 5.5, 6.0, 6.5, 7.0, 7.5, 7.75, 8.0, 8.25, 8.5 and 9.1. Purified GFPmut3 and mCherry were both added to a final concentration of 1 μM in each of the buffers. 100 μl aliquots of these were then transferred to a 96 well microplate (NUNC), and then placed in a FLUOROstar Optima (BMG LabTech) where the GFP (excitation 480/10 nm; emission 520/10 nm) and mCherry (excitation 570/10 nm; emission 620/10 nm) signals were read before being processed in Microsoft Excel.

## Results and Discussion

In this work we set out to study the recruitment of transcriptional machinery into the spore during sporulation using mCherry labelled RNA polymerase (RNAP; in-frame fusion to the β' subunit, Table 1) and GFP labelled transcription factor NusA, along with small auxiliary RNAP subunits δ, ω and YkzG (Table 1). A trend was noticed that showed very little RNAP-mCherry fluorescence, but a high amount of GFP fluorescence of tagged NusA during stages III to V (refer to Figure 1) of sporulation. This was followed by a complete reversal of fluorescence in the final stages when the spore became phase bright, with high RNAP-mCherry, and almost undetectable NusA-GFP signals. Similar results were obtained when co-localising mCherry labelled RNAP (β' subunit) with GFP tagged RpoE (RNAP δ subunit), YloH (RNAP α subunit) and YkzG (uncharacterised RNAP subunit).

**Figure 2:**
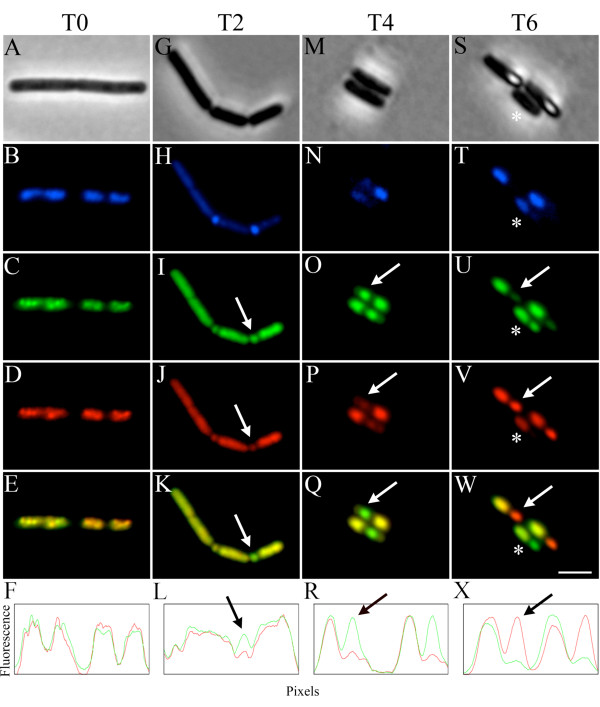
**Stage-specific fluorescence of GFP and mCherry**. The stage specific fluorescence of RNA polymerase subunits β and β' during sporulation. Panels A, G, M and S are phase contrast images, Panels B, H, N and T are DAPI stained DNA images, Panels C, I, O and U are the β-GFP images, Panels D, J, P and V are the β'-mCherry images, Panels E, K, Q and W represent image overlays of the respective β-GFP and β'-mCherry images, and Panels F, L, R and × are linescans taken through the respective image overlays with green lines representing β-GFP and red lines representing β'-mCherry. The linescan in panel L is taken through the two asymmetrically dividing cells on the right of panel K. The lane scan in panel X is taken through the two cells with phase bright spores in panel W. The white arrows in the micrographs correspond to the black arrow in the respective linescan. The asterisks in the T6 micrographs are discussed in detail in the text. Fluorescence is in arbitrary units. Scale bar is 2 μm.

To further investigate this, we labelled RNAP with both mCherry and GFP to determine if the spectral properties of GFP and mCherry were affected at the different stages of sporulation. Prokaryotic RNAPs are highly conserved comprising four essential subunits; two α subunits, a β and a β'. We created EU156 (Table 1), which is a strain containing a GFP fusion to the β subunit and an mCherry fusion to the β' subunit and observed the localisation patterns during sporulation. Results are presented in Figure 2, showing phase contrast (top panels), DNA (blue), β'-mCherry (red), β-GFP (green), an image overlay of the β'-mCherry and β-GFP signals and a linescan taken through the image overlay. Images were taken every two hours from vegetative growth (T0) through to stage V-VI of sporulation (T6).

It is clear from the linescan during vegetative growth that the fluorescence of both β-GFP and β'-mCherry are equal and consistent with what would be expected when subunits of equal stoichiometry are labelled (Figure 2F). Because the sporulating culture was asynchronous and not all cells go on to sporulate, the T2 images show cells that are stage 0 or I (the two cells on the left), and those in stage II (the two cells on the right). Although the GFP and mCherry fluorescence is quite similar between cells at T2, a slight drop in mCherry fluorescence can be seen in one of the developing forespores (arrows in Figure 2I-L), which by reference to the DAPI stained DNA image (Figure 2H), looks to be at a later part of stage II than the left cell (stage II_ii _*vs *II_i_; Figure 1). By T4 there is a dramatic difference in fluorescence between GFP and mCherry, with almost no detectable mCherry in the developing spore, and bright GFP fluorescence (arrows in Figure 2O-R). At T6 when sporulation reaches stage V-VI and developing spores become phase bright, there is a complete reversal of this fluorescence pattern. The fluorescence from the β-GFP becomes almost undetectable, while the fluorescence from β'-mCherry returns to levels similar to that seen in the mother cell (arrows in Figure 2U-X). Interestingly, due to the asynchrony of sporulation, there is a cell in this micrograph still with a phase dark spore (indicated by the asterisks) and fluoresces similar to that described for cells at stage III-IV seen in T4 cultures.

**Figure 3:**
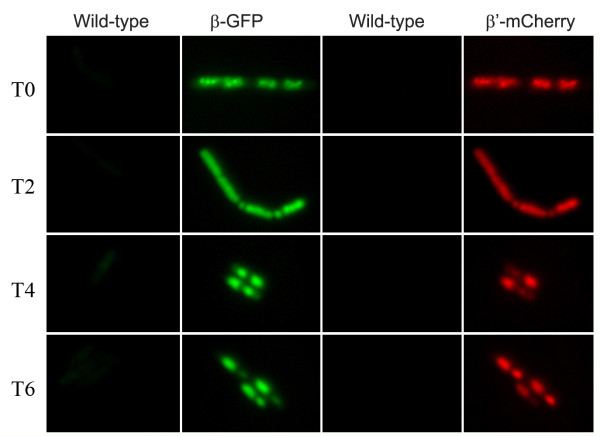
**Autofluorescence during spourulation**. Autofluorescence is very low during sporulation. Fluorescence emission signals for wild type (left) and fluorescent fusion strains (right) are shown for the GFP and mCherry channels at the time (hours) after resuspension into sporulation medium. The images have been equalised so that the fluorescence through the GFP channels is identical for the wild type and fluorescent fusion strain, and likewise in the mCherry channel. The cells for the GFP and mCherry fluorescent fusions are the same as those shown in Figure 2 for reference.

The pattern of fluorescence described above was also observed for β'-mCherry with GFP labelled NusA, ω, δ and YkzG, as well as YkzG-mCherry/NusA-GFP, YkzG-mCherry/β'-GFP and NusA-mCherry/YkzG-GFP pairs (Table 1; data not shown). Regardless of the protein of interest, GFP always fluoresced brighter during the initial stages of sporulation before dropping off in phase bright spores, while mCherry always fluoresced less brightly in the initial stages before fluorescing brightly in phase bright spores. Fluorescence microscopy was performed on the wild-type parent strain 168trp+ at the same time as the fluorescent protein data using identical image acquisition and processing settings. Results indicated there was virtually no auto-fluorescence using our GFP and mCherry filters that could account for this phenomenon (Figure 3).

**Figure 4:**
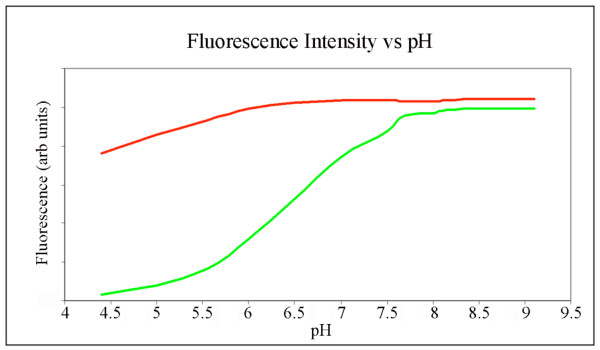
**The pH-dependent emission profile of GFP and mCherry**. GFP is more sensitive to pH change than mCherry. The pH-dependent emission profile of GFP and mCherry was investigated by resuspending GFP and mCherry in phosphate buffers with a pH ranging from 4.4 to 9.1. The emission was then plotted against the pH value to obtain the profile.

It was previously shown that pH fluctuations occur during sporulation of yeast and *Bacillus sp*. with vegetative cells and the mother cell generally having a pH of around 8, while the dormant spore has a pH of around 6 [9-11]. A study monitoring the internal pH changes during sporulation of *B. megaterium *has shown that the pH in the spore remains constant for the first four hours, probably to around late stage III or stage IV, before rapidly dropping over the following two hours [11]. To investigate whether local changes in pH during sporulation could account for the altered intensity profiles of GFP and mCherry, the pH-dependent emission profiles of these fluorescent proteins were determined and are shown in Figure 4. These profiles suggest that the fluorophore of mCherry is more tolerant to a drop in pH when compared to GFP. Indeed an internal pH of below 6.5 in the phase bright spore could very well explain the fluorescence pattern seen in Figure 2 for phase bright spores (Figure 2S-X). However, nowhere in the intensity profile is there a pH where the mCherry emission is adversely effected while GFP emission is unaffected.

Both GFPmut3 and mCherry have similar maturation times (around 30 minutes for GFPmut3 [2] and between 15 and 40 minutes reported for mCherry [12,13], so a slower maturation time of mCherry is unlikely to explain the reduced fluorescence observed during the early stages of sporulation. One of the major differences in the maturation process is the need for two moles of molecular oxygen to form a mature mCherry chromophore, compared to the one mole required for GFP [3,14]. It is feasible that a reduction in oxygen availability could account for these emission differences during sporulation, although it was recently found that mCherry maturation was unimpeded under hypoxic conditions when expressed in *Mycobacterium tuberculosis *[15].

We believe the results presented here have far reaching implications on the use of fluorescent proteins to quantitatively study gene regulation during live cell imaging involving intracellular physiological changes, or even between intracellular compartments in 'steady state' eukaryotic cells. Indeed a very recent publication on gene expression using GFP and mCherry reporter fusions appeared to identify exactly this phenomenon. The expression of *kinA*, an important kinase involved in the phosphorelay pathway during sporulation was found to be expressed much earlier, with a larger peak when the *kinA *promoter was fused to *gfp *when compared to the promoter fusion to *mCherry *[16]. The authors suggested this could be due to a slower maturation time of mCherry compared to GFP, however, as mentioned above the documented maturation times for these fluorescent proteins are similar, and is therefore unlikely to explain the results observed during that work.

In summary, we have identified artefacts that may arise when quantitatively using GFP and mCherry during sporulation. It appears that the emission profile of GFP is not substantially affected during early sporulation, and is probably the most effective fluorescent protein for this phase in sporulation before the pH drops at around stage IV-V of sporulation. Conversely, this period appears to adversely affect mCherry emission, but it recovers in the final stages of sporulation. Considering sporulation is a paradigm in which to study gene regulation and expression, we urge caution when interpreting fluorescent protein reporter results. As Remington pointed out in a review in 2007, "the lack of understanding of the maturation, photochemistry and photophysics of fluorescent proteins can lead to significant pitfalls in everyday applications" [14].

## Competing interests

The authors declare that they have no competing interests.

## Authors' contributions

PL and GD designed experiments. GD and KB performed experiments. GD and PL interpreted results and prepared manuscript for publication. All authors read and approved the final draft.
